# Neurodevelopmental evaluation of children who were operated due to congenital anomaly with the ‘Ages and Stages Questionnaire (ASQ)’ and ‘ASQ: Social–Emotional’

**DOI:** 10.1007/s00383-023-05625-5

**Published:** 2024-02-01

**Authors:** Bilge Turedi, Gulnur Gollu, Ufuk Ates, Kenan Kose, Ozgur Oner, Meltem Bingol-Kologlu, Aydin Yagmurlu, Tanju Aktug, Huseyin Dindar, Murat Cakmak

**Affiliations:** Ankara University, Ankara, Turkey

**Keywords:** Anorectal malformation, Congenital anomaly, Developmental delay, Intestinal atresia, Surgery

## Abstract

**Background:**

The purpose of surgeries performed for congenital anomalies in children is to increase the survival rates and provide a developmental comparison to that of their peers.

**Aim:**

The objective of this study was to investigate the development of children following surgery for congenital anomalies and the risk factors affecting their development.

**Methods:**

Our study included 33 children who underwent surgery for gastrointestinal anomalies in our clinic between 2011 and 2016, and did not have any syndrome, chromosomal abnormality, or additional abnormality. Developmental levels were evaluated using the Ages and Stages Questionnaire (ASQ) and the ASQ: Social–Emotional (ASQ: SE) scales adapted for the use on Turkish children. Data on patient history were obtained retrospectively from patient files.

**Results:**

The study included 33 patients, including 11 with esophageal atresia, 6 with intestinal atresia, 11 with anorectal malformation, and 5 with Hirschsprung's disease. Developmental delay was found in the ASQ of 72.7% of the patients and the ASQ: SE tool was 27% of the patients. The rate of patients with scores below the threshold from each parameter of ASQ was higher than that of the normal population (*p* < 0.05). Development delay was detected using the ASQ scale in 100% of those with microcephaly at birth, in 91% of premature infants born between 1500 and 2500 g, and in 83.3% of those with low birth weight to gestational age.

**Conclusions:**

In children who underwent surgery due to congenital anomalies, an evaluation through developmental tests, a post-surgical follow-up process, and a referral to the relevant disciplines when necessary may increase the success of surgery as well as increase the life quality of the patient.

## Background

Among all operations in pediatric surgery practice, there are a considerable number of operations performed due to congenital anomalies. Most of them are diseases of unknown etiology and are thought to develop genetically. Congenital anomalies are associated with problems of varying degrees of severity depending on the affected area [1]. One of the most frequently affected areas is the gastrointestinal (GI) system, with a rate of 1.3 per 1000 live births, and these anomalies typically manifests itself after birth [2, 3]. Although surgical treatment is frequently applied for congenital GI system anomalies, the effect on patient neurological or social–emotional development in the post-treatment period is not understood well [4]. Various questionnaires have been used to evaluate the neurological and social-emotional development in children. The Ages and Stages Questionnaire (ASQ) and ASQ: Social–Emotional (ASQ: SE) tool have been frequently used and can be easily applied by families [5]. While the ASQ scale has been used to evaluate neurodevelopment in five domains (communication, gross motor skills, fine motor skills, problem solving, and personal-social), ASQ: SE scale has been used to evaluate social and emotional development [6, 7].

The main purpose of surgery in patients with congenital anomalies is to increase survival rates, though it is also important to reduce morbidity rates and provide neurological and 'social–emotional' development comparable to their healthy peers. This study was aimed to better understand the neurodevelopmental and social–emotional developmental levels in children that underwent surgery for congenital GI anomalies. In addition, our study was aimed to investigate the risk factors affecting the neurological and social–emotional developments in these patients.

## Methods

The study was conducted retrospectively with the approval of the institutional ethics committee, dated 14 September 2015 and numbered 14-595-15, in accordance with the declaration of Helsinki. Informed written consent was obtained from each child's legal heir.

**Fig. 1:**
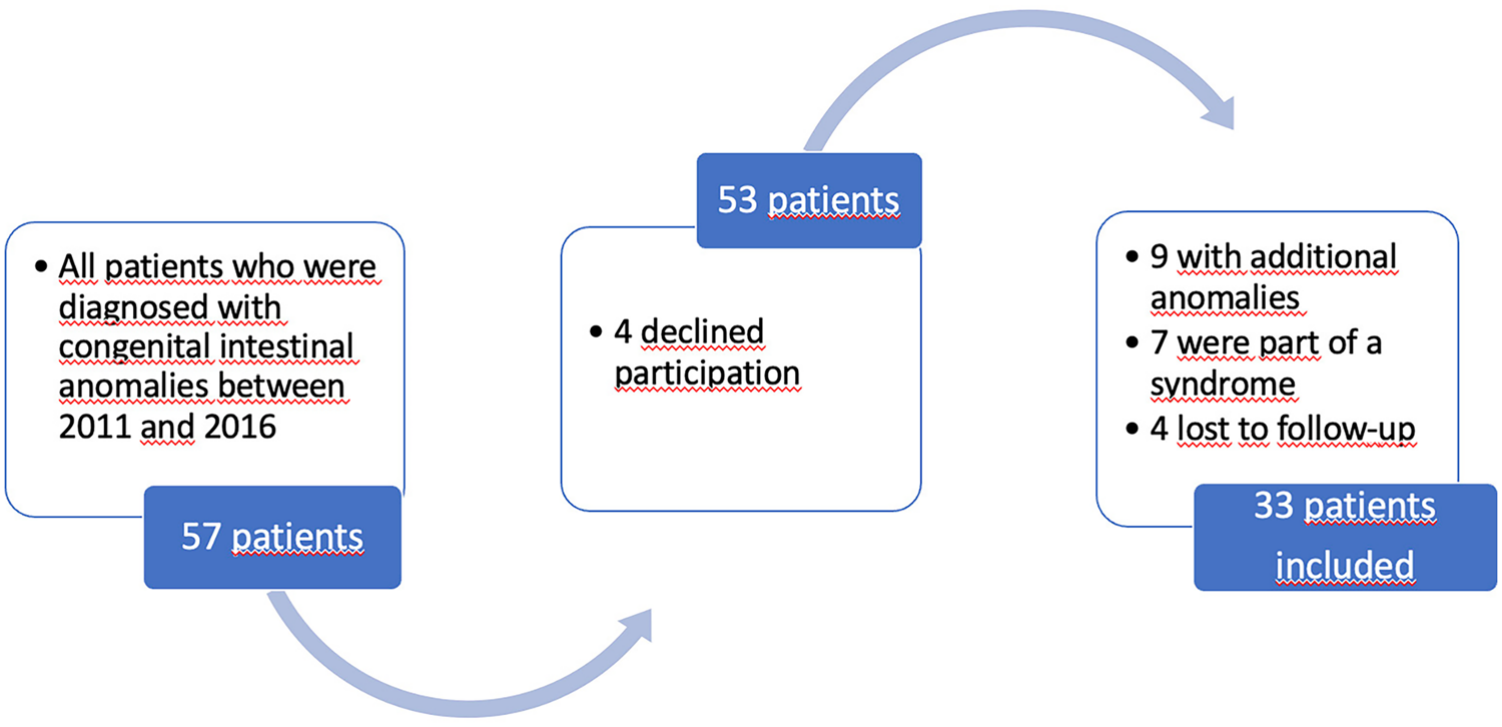
Flow diagram of the study design

The study included a total of 33 patients that underwent surgery for esophageal atresia (EA), intestinal atresia (IA), anorectal malformation (ARM), and Hirschsprung's disease (HD) between 2011 and 2016. Children with syndromic appearance, chromosomal abnormality, or additional abnormalities were excluded. To avoid selection bias, all patients born with congenital intestinal anomalies between 2011 and 2016 in our center were randomly reviewed and patients who met the inclusion criteria formed the study group as shown in the flow diagram of the study (Fig. 1). Data including gestational age of the patients, age of enrollment, gender, birth weight, head circumference, and prenatal risk factors (i.e., maternal drug use, smoking status and history of infections, eclampsia-preeclampsia, and surgeries) were obtained retrospectively from patient files. The adjusted age of the premature patients was calculated by Squires et al. scale adjusted for premature children between 4 and 24 months [8]. Data on the family's economic and educational status were obtained through interviews with the parents. As the poverty threshold was accepted as 4,700 Turkish Lira by the Turkish Statistical Institute at 2016, we classified the families of patients accordingly.

In our study, we used ASQ and ASQ: SE tools, which were adapted for Turkish children by Kapçı et al. [9] and Küçüker et al. [10], respectively. The ASQ scale was used to conduct a neurodevelopmental assessment. It contains 19 sub-questionnaires which are suitable for each age group (in terms of months) between 4 and 60 months. This scale included the domains of communication, fine motor and gross motor skills, problem solving, and personal-social development. Each domain consisted of six questions with three answer options: “yes”, “sometimes”, and “not yet”, which were scored as 10, 5, and 0, respectively. A threshold score was determined for each domain. If the score from each domain was above this threshold, it was interpreted as a normal result. Patients with scores below the threshold from one or more domains were referred to a relevant specialist. The ASQ: SE scale was used to conduct a social and emotional developmental assessment. In this scale, there were 29 questions with four answer options: “often or always”, “sometimes”, “rarely or never”, and “check if this is a concern”. These were scored as 0, 5, 10, and 15, respectively. Those who scored higher than the threshold score of 67.5 in total were directed to a relevant specialist [6, 7].

During outpatient clinic examinations in the routine follow-up process, we preferred ASQ and ASQ: SE tools for our patients at different age periods that was appropriate for their age group. These questionnaires are based on direct observation of the parents and are easily filled out by the parents themselves. In addition, they are the only screening scales with proven validity and reliability in Turkey. The parents filled in the scales appropriate for the age of the child (in months) under the supervision of a single physician in a quiet room suitable for interview. Questionnaires were handed over to the parents so that questions inquiring about previously untested or not currently observed skills could be answered at home under natural conditions. The results of the neurodevelopmental and social–emotional developmental scales were statistically compared with the data obtained from the normal population of comparable age group by Kapçı et al. [9] and Küçüker et al. [10].

## Statistical analysis

Data were analyzed using the Statistical Package for the Social Sciences (SPSS) 22.0 (IBM Corporation, Armonk, New York, United States). One-sample T-test was used to compare categorical variables. The categorical data were expressed as n (number) and percentage (%). A p value of less than 0.05 was considered as significant.

## Results

Of the 33 patients included in the study, 17 (51.5%) were male. Eleven patients were operated for esophageal atresia, six for intestinal atresia, five for Hirschsprung’s disease, and 11 for anorectal malformation. The mean age of all patients was 24 months, which was 12 months in the esophageal atresia group, 23.5 months in the intestinal atresia group, 28.6 months in the Hirschsprung's disease group, and 16.9 months in the anorectal malformation group. Twenty of the patients were born at term (37 weeks) and 13 were born prematurely (< 37 weeks). One of the patients had extremely low birth weight (< 1000 g), one had very low birth weight (< 1500 g), 14 had low birth weight (< 2500 g), and 17 had normal birth weight (2500 g). Head circumference at birth was normal in 20 patients, whereas it was above the 90th percentile in 7 patients and below the 10th percentile in 6 patients. Prenatal risk factors included a history of maternal infections in one patient, smoking in one patient, and eclampsia-preeclampsia in one patient. Among the parents, 11 fathers (30.3%) and 7 mothers (21.2%) were university graduates. The families of 29 (88%) of the patients had an income below the poverty level (Table 1).

**Table 1:** General characteristics of 33 patients included in the study

	Number of patients *n* (%)
Gender	
Male	17 (52%)
Female	16 (48%)
Anomalies	
Esophageal atresia (EA)	11 (33%)
Intestinal atresia (IA)	6 (18%)
Hirschsprung’s disease (HD)	5 (16%)
Anorectal malformation (ARM)	11 (33%)
Gestational age	
≥ 37 weeks	20 (61%)
< 37 weeks	13 (39%)
≥ 2500 g	17 (52%)
< 2500 g	16 (48%)
Birth weight	
1500–2500 g ≤ 1000 g	14 (88%)
1000–1500 g	1 (6%)
≤ 1000 g	1 (%6)
10–90th percentile	20 (61%)
Head circumference	
> 90th percentile	7 (21%)
< 10th percentile	6 (18%)
Infections	1 (3%)
Prenatal risk factors	
Eclampsia–preeclampsia	1 (3%)
Smoking	1 (3%)
Parental educational status	
Father with university degree	10 (31%)
Mother with university degree	7 (21%)
Income status	
> Poverty level	4 (12%)
< Poverty level	29 (88%)

*ARM* anorectal malformation, *EA* esophageal atresia, *HD* Hirschsprung’s disease, *IA* Intestinal atresia

The ASQ detected developmental delay in 24 patients (72.7%) and ASQ:SE tool in nine patients (27%) (Table 2). The rate of patients with developmental delay was found to be higher than the normal population (*p* < 0.05).

**Table 2:** Distribution of the results of ASQ and ASQ: SE scales by disease groups

Congenital anomalies		Total, *n* (%)	EA (*n*)	IA (*n*)	HD (*n*)	ARM (*n*)
	Normal developmental parameters	9 (27%)	3	1	–	5
ASQ	Delay in 5 parameters	4	2	1	–	1
	Delay in 4 parameters	4	1	–	1	2
	Delay in 3 parameters	6	3	1	2	–
	Delay in 2 parameters	5	–	2	1	2
	Delay in 1 parameters	5	2	1	1	1
	Total number of patients with delayed parameters	24 (73%)	8	5	5	6
ASQ-SE	Normal social–emotional developmental parameters	24 (73%)	9	4	1	10
	Social-emotional development should be evaluated	9	2	2	4	1

*ARM* anorectal malformation, *ASQ* ages and stages questionnaire, *ASQ-SE* Ages and Stages Questionnaire Social–Emotional, *EA* esophageal atresia, *HD* Hirschsprung’s disease *IA* intestinal atresia

The ASQ scale found developmental delay in communication (42.4%), gross motor (45.5%) and fine motor skills (39.4%), problem solving (45.5%), and personal–social skills (36.4%) domains of the patients. The rate of patients with developmental delay in each domain was found to be higher when compared with the reference rates (*p* < 0.05) (Table 3).

**Table 3:** Comparison of patients with growth delay using ASQ scale domains with reference rates

	Developmental delay, *n* (%)	Reference values *, %	*P*^#^
ASQ—communication	14 (42)	15.8	< 0.001
ASQ—gross motor skills	15 (46)	18.0	< 0.001
ASQ—fine motor skills	13 (39)	24.4	0.045
ASQ—problem solving	15 (46)	25.2	0.007
ASQ—personal-social	12 (36)	20.1	0.020

*Squires J, Potter L, Bricker D. The ASQ User's Guide for the Ages and Stages Questionnaires: A Parent-Completed, Child-Monitoring System. 2nd ed. Baltimore: Paul H. Brookes Publishing Co.; 1999

^#^*P* values of comparison percentages by one-sample *T*-test

Evaluation of each congenital anomaly with the ASQ scale found developmental delay in eight (72.7%) patients with esophageal atresia, five (83.3%) patients with intestinal atresia, five (100%) patients with Hirschsprung's disease, and six (54.5%) patients with anorectal malformation. The rate of growth delay was found to be higher when compared with the normal population (*p* < 0.05). Furthermore, the ASQ:SE tool found developmental delay in two patients with esophageal atresia (18.2%), two patients with intestinal atresia (33.3%), and one with anorectal malformation (9.1%). However, these rates were not statistically different from those in the normal population (*p* > 0.05). Four of five patients (80%) with Hirschsprung's disease had developmental delay, which was significantly higher than the normal population (*p* < 0.05).

The ASQ and ASQ:SE tools detected developmental delay in 10 (91%) and 4 (36.4%) of 11 patients born prematurely with low birth weight (between 1500 and 2500 g), respectively. Five of six patients (83.3%) with low birth weight for gestational age had developmental delay on the ASQ scale and three (50%) on the ASQ:SE scale. In addition, all of six patients (100%) with a head circumference below the 10th percentile had developmental delay on the ASQ and ASQ: SE scales. Sixteen of the patients (48.4%) had microcephaly and/or low birth weight. In addition, 93.7% of the children, who had at least one of these two conditions, had a developmental delay.

## Discussion

In our study, the neurodevelopment and social–emotional development of children who underwent surgery for congenital GI system anomalies were evaluated using ASQ and ASQ: SE developmental scales which are adapted for Turkish children [9, 10]. We achieved higher rates of developmental delay in this group of children compared to the normal pediatric population.

Children born with congenital anomalies are considered potential candidates for neurodevelopmental delay, regardless of the type of anomaly [11]. Studies have reported that the postoperative developmental and psychosocial process in these patients is just as important as the postoperative surgical follow-up process [12]. For this reason, developmental follow-ups of these children, as well as surgical follow-ups in the postoperative period, are gaining importance for us, pediatric surgeons. There are many factors that affect the neurodevelopmental status change in follow-up. Among the leading ones are the socioeconomic status of the family and the knowledge level of the mother. It is well established that educated parents provide a higher rate of positive contribution to the neurodevelopment of their children [13]. In 2007, Ertem et al. presented a study which aims to evaluate the maternal knowledge level on the development of their children and reported an insufficient level of maternal knowledge. They argued that the developmental parameters of these children are directly related to the level of the maternal knowledge of child development [14]. Reich et al. reported increasing maternal knowledge about normal child development would be beneficial for children [15]. In addition to the education levels of the mothers, it is thought that the level of knowledge about normal child development is also effective in this process. In our study, we do not have data on the knowledge levels of mothers about normal child development, but we evaluated the data on their educational status. Only 21.2% of the mothers participating in this study were found to be graduates of higher education. This situation may have related to the low scores obtained by the children in our study.

Etiological studies emphasized that socioeconomic level is another possible factor affecting it. There are studies in the literature to reveal the relationship between socioeconomic level and income and fetal growth delay. Potijk et al. reported that prematurity and low socioeconomic status are two important risk factors for neurodevelopmental delay in their study which evaluated 926 preterm and 544 term babies using the ASQ scale. And additionally, they reported the rate of fetal growth delay is 3–9% in high-income countries, while this percentage is six times higher in low-income countries [16]. Similarly, another study reported more motor developmental delay in children of families with low socioeconomic status [17]. Due to the region where our hospital is located, the patient profile mostly consists of families with low socioeconomic levels. We wanted to investigate whether the socioeconomic level of our patients' families would be a factor affecting their children's development, and we found that children with delayed developmental parameters were more likely to come from families with low economic levels. In our country, state support is provided to children with some serious diseases upon official application, but there is no routine support that families in this patient group receive from the state. Knowing that the socioeconomic level of families also affects the development of their children is of great importance for us, pediatric surgeons who follow these patients, to help them get support. In our study, we found high rates of developmental delay in patients having parents with low socioeconomic status. Although this result is not the only factor affecting the development of children, it is thought to be important in terms of emphasizing that low socioeconomic level may have also been effective in the results.

It was also investigated that congenital anomalies do not only affect the relevant organ, but they can also affect the fetus in every aspect, therefore these problems may be predicted with some factors depending on specific measurements. Considering the studies investigating these etiological factors in the literature, head circumference was thought to be a predictive measurement for developmental delay and cognitive problems [12]. Studies have reported that children with small head circumference measurement may encounter neurocognitive problems more often and should be followed more closely [15]. Although this measurement alone is not sufficient to clearly explain the results, it is thought to be one of the factors that may affect it. In our study, developmental delay was detected in all patients with a head circumference below the 10th percentile. Considering that there are other modifying factors, it is thought that the measurement of head circumference in these children can be useful for predicting developmental delay or cognitive problems when evaluated together with other parameters.

Low birth weight is thought to be one of the possible etiological factors that have been investigated. Aite et al. evaluated 155 children who were operated for congenital anomalies (41 of whom were operated due to esophageal atresia) by using the Bayley Scales of Infant and Toddler Development. The third edition (Bayley-III) at one year of age. They found and reported that low birth weight may be a potential risk factor for motor and cognitive developmental delay [17]. In another study investigating the effect of other risk factors, prematurity was found to be an affecting risk factor that enhances the rate of developmental delay [18]. Although it is known that prematurity may cause this on its own, we investigated what effect it would have on the results of our study group, which consisted of patients who were operated for congenital anomaly. We found a higher rate (91%) of developmental delay in patients born prematurely with a birth weight of 1500–2500 g. The most ideal way to detect the etiologic cause was to compare the results of premature births that did not undergo surgery and low birth weights. But in our study, we aimed to reveal the possible effects on our own postoperative patients. We also believe that premature birth when in association with low birth weight might be one of the risk factors for developmental delay in our patients.

Our patients in our study consisted of 4 groups who were operated for different congenital anomalies. When the literature is reviewed in terms of anomaly type, it is possible to come across studies performed on patients who have been operated for different anomalies. In a study including 40 children who had been operated for congenital GI system anomalies, neurodevelopmental retardation and impaired language development were reported [4, 11, 19]. There are also studies involving children who have been operated especially for esophageal atresia. In 2010, Hamrick et al. reported that children that underwent surgery for esophageal atresia had a fivefold higher need for special education and a two-fold higher rate of behavioral and emotional delay [20]. Similarly, Bouman et al. suggested that these patients have serious emotional and behavioral problems that require special education and a need for neurodevelopmental follow-up [21]. In 2013 Holden et al. reported normal mental development in the patients with anorectal malformations (ARM), but motor developmental delay in 13% of the patients [23]. Also in 2014, Bevilacqua et al. reported poor cognitive and motor skills in this patient in the evaluation of the development of children operated on for intestinal atresia and anorectal malformation [22]. More et al. investigated the developmental status of 54 children operated for Hirschsprung's disease, using the Griffiths Mental Development Scales (GMDS) and reported lower scores compared to the normal population in their study. However, they reported a developmental level within normal limits [25]. In our study, we reported higher rates of neurodevelopmental delay in the EA group, but similar rates of social–emotional developmental delay compared to the normal population. Considering the HD group, the ASQ: SE scores were found to be lower than the other groups. According to our results, it can be interpreted that the OA group should be followed more closely in this respect due to ASQ scale and the HD group should be supported more due to ASQ: SE scales, but the level of evidence will be low due to the small number of patients.

On the other hand, a study involving 20 children from a similar group reported that some of these patients had low cognitive and motor skill scores at the sixth month but achieved normal scores at the age of one year [24]. Due to the limited working time, it cannot be applied to all patients consecutively at different developmental months in our study. For more optimal evaluation, we think that repeating the scales consecutively in different developmental months will facilitate the interpretation of the findings.

There are some limitations of the study. First of all, the developmental levels of the patients were evaluated only with scales. There are some factors such as ventilation period, presence of hypoxia, presence of complication, anesthesia period, delay of nutritional support, time of TPN start. Evaluation of these parameters could help discussing the results more clearly. To avoid the possible artifacts in immediate postoperative period that may affect the results, first evaluation was made after a minimum 6-month time distance to last surgery. The second limitation of our study is the relatively low number of patients. These congenital anomalies of 4 different groups have a relatively low incidence and are not frequently seen in surgical practice. The third limitation is the absence of a control group. As a result, the developmental measurements consisting normal population were absent and we could not be able to point out the comparative results with the normal population. The study group contains only children who were operated for congenital anomaly so we did not have the comparison of results of premature infants without congenital anomaly with similar prematurity levels. Another limitation of our study is that, due to its cross-sectional nature, it can show the relationships between factors that may be effective, but cannot make definitive comments about causal origins. However, we are of the opinion that these limitations do not have a high impact on our results. We do believe that there is a need for further large-scale studies on this subject.

To our knowledge, this is the first study in the literature which aims to evaluate the neurodevelopmental status of children who underwent surgery for different congenital GI system anomalies with an average age of 2 years using the ASQ and ASQ: SE developmental scales. The study group which consists of children of parents with low socioeconomic status, makes our study different from other existing studies.

## Conclusion

It should be kept in mind that the children who are operated on due to congenital GI system anomalies are at high risk not only for poor postoperative results but also for neurodevelopment and social–emotional developmental delay. Patients with risk factors such as microcephaly, low birth weight for gestational age, prematurity, and low socioeconomic level should be closely monitored for neuromotor, social, and emotional development. These patients should not only undergo a postoperative surgical follow-up but also an assessment using developmental scales. Thus, timely referral to the relevant disciplines will ensure a healthy developmental process and increase the life quality of the patients who undergone surgical procedures for congenital anomalies.
